# Does General Motivation Energize Financial Reward-Seeking Behavior? Evidence from an Effort Task

**DOI:** 10.1371/journal.pone.0101936

**Published:** 2014-09-26

**Authors:** Justin Chumbley, Ernst Fehr

**Affiliations:** Department of Economics, University of Zurich, Zurich, Switzerland; University of Pennsylvania, United States of America

## Abstract

We aimed to predict how hard subjects work for financial rewards from their general trait and state reward-motivation. We specifically asked 1) whether individuals high in general trait “reward responsiveness” work harder 2) whether task-irrelevant cues can make people work harder, by increasing general motivation. Each trial of our task contained a 1 second *earning interval* in which male subjects earned money for each button press. This was preceded by one of three predictive cues: an erotic picture of a woman, a man, or a geometric figure. We found that individuals high in trait “reward responsiveness” worked harder and earned more, irrespective of the predictive cue. Because female predictive cues are more rewarding, we expected them to increase general motivation in our male subjects and invigorate work, but found a more complex pattern.

## Introduction

Several classical psychological theories assume that two basic brain systems motivate behavior: one responds to potential punishment/frustration, the other to potential reward/relief [1], [2], [3], [4]. Despite recent variations to this idea [5], an underlying “reward system” is still widely thought to influence individual differences in behavior [6], [7], [8], neurophysiology [9], and personality [10]. A central property of this “reward system” is that it energizes reward-seeking behavior. We therefore measured the energy of reward-seeking behavior in terms of *the rate of work for financial rewards* and aimed to predict this from subjects' trait and state reward motivation. We measured the former with a standard questionaire measure of “reward responsiveness” [11].

Regarding the latter, our question was whether incidental cues could increase general motivation, driving subjects to work harder. Previous work has shown that incidental sexual cues alter people's goal-directed choice behavior [12], [13], [14]. We wondered whether task-irrelevant sexual cues could also influence general motivation to work for seperate financial rewards in a task *without* discrete choices. This question arises from classical empirical work [15] and recent theoretical work [16] which has documented two aspects of motivation. The first type is directed towards achieving a specific goal. The second, less intuitive, aspect is a general level of invigoration: such motivation should non-specifically increase work, even in a task without discrete choices, i.e. not a standard “decision-making task”.

**Figure 1:**
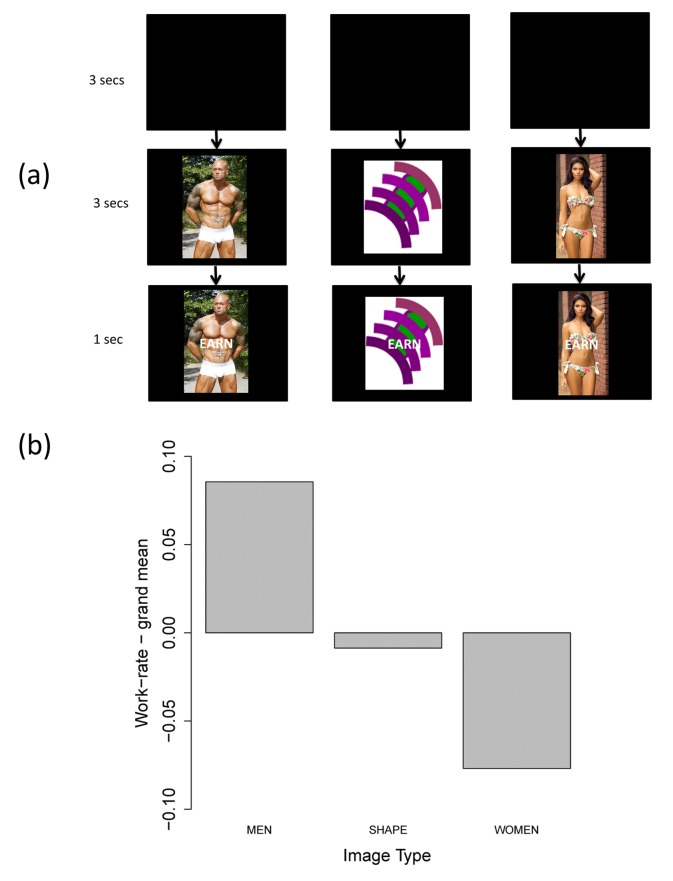
Different cue types and how they influenced work-rate. Figure 1a. This gives an example trial for each of the three types of cue: female, male and shape cues. Figure 1b. Work-rate following male, female and shape anticipatory cues, relative to average work-rate. Source: Bbpics, ShareAlike 3.0 Unported, https://commons.wikimedia.org/wiki/File:Male_Model_John_Quinlan_in_Calvin_Klein_Low-Rise_Boxer_Briefs.JPG Source: earthlydelights, Bandeau Bikini adjusted, CC-BY 2.0, https://www.flickr.com/photos/earthlydelights/4423552169/.

In our task, subjects main “choice” was not between discrete alternatives, but how vigorously to respond with a given, rewarded behavior [16]. Male subjects intermittently had the opportunity to earn money for each button press, approximately 5 US cents per press (0.05 CHF). This opportunity was signaled by predictive cue, which was incidentally a female erotic cue, male erotic cue or abstract shape, see Figure 1a. We have elsewhere shown that female cues are more subjectively rewarding. Here we asked whether they increase work-rate, as would be predicted if they were generally invigorating [15], [16]. We further asked whether greater trait reward-responsiveness, as measured by questionaire, would predict greater of interference of incidental reward cues on instrumental responses for money.

## Methods

### 0.1. Experiment

#### 0.1.1. Subjects

The experiment was conducted in a computer laboratory at the University of Zurich. A total of 52 subjects (18–30 years old, all male) were tested in four sessions. The study was approved by the Human Subjects Ethics Committee, Dept. of Economics, Zurich. Subjects provided written consent according to a procedure approved by the Human Subjects Ethics Committee. Subjects were not deceived in any part of this study. Subjects' payments depended on their real performance and choices in the task.

#### 0.1.2. Procedure

Subjects were welcomed into a reception hall. Having been identified and instructed of the ground rules (see below), they were conveyed *en masse* into a separate behavioral lab, where they were each randomly assigned to an isolated computer booth. Subjects could only see their own screen, and communication was prohibited. They were first given written and verbal instructions, as follows.

- Whenever you see the word “EARN” on the screen, you can earn 5 centimes simply by pressing the space bar. You can press as often as you want whenever “EARN” is on the screen: you will always earn.
- You will not earn anything for pressing the space bar when the word “EARN” is not on the screen. You will never lose money.
- Try to earn as much money as possible.
- You will see photographs and images on the screen about this task, but these are not relevant to the task and you should ignore them.

The experimenter then left the room.

We independently varied the type of images across trials. There were 3 types of images: 10 MEN, 10 WOMEN and 10 FRACTALS. Pictures of men and women were cropped from head to thigh and featured semi-nude models (in underwear) posing in provocative body postures. Pictures of fractals were abstract, meaningless shapes. To obtain copies of these images, please contact the corresponding author. In previous work, we have shown that male subjects on average find female images more rewarding: they express a preference for viewing female images. Each image was presented four times, giving 120 trials. The order of presentation was randomized across subjects. Each image was presented for exactly 4 seconds per trial. After 3 seconds, the word “EARN” was presented, for exactly 1 second. There was then a inter-trial interval of 3 seconds. Subjects then completed the ARES personality questionnaire [11], before being payed and dismissed.

To characterize subjects self-reported “reward responsiveness”, we used a sub-scale from a widely used personality measure, ARES BIS/BAS [11]. Please see supplementary material for details on reliability of ARES and validity of BAS more generally. In general, this personality questionnaire aims to measure two behavioral systems that are tightly coupled to subjective emotional experience [11]: a behavioral inhibition system (BIS) and a behavioral activation system (BAS). BIS I measures anxiety and BIS II, frustration. BAS I contains questions evaluating the drive behind goal-directed behavior. BAS II measures the responses to reward attainment. We used the short version of the ARES-scales which contains 20 items from [11]. The English version is provided in the supplementary material. BAS I and BAS II resemble “drive” and “reward responsiveness” respectively, in Carver & White's BAS scales [17], [18]. This questionnaire does not include a scale corresponding to “fun seeking”, which is less straightforward to derive from biobehavioral models of animal reinforcement sensitivity and may relate more to impulsivity [18].

The BAS II subscale quantifies reward responsivity with five items (the final two are scored in reverse). This resembles BAS II – “reward responsiveness” – in Carver & White's BAS scales [17], [18]. These five items are…

- Even small things make me really happy.
- I am easily delighted.
- It makes me very happy to achieve a goal I strove for.
- I get rather seldom really excited about something.
- I rarely get excited, even when I get something that I really wanted.

#### 0.1.3. Statistical analysis

Our analysis asked whether work depended on subjects' BAS II “reward responsiveness” 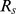 (between-subject) and incidental cue type (within-subject). To jointly address these within- and between-subject hypotheses, we used a multilevel, generalized linear mixed model to explain the number of button-presses on each trial. Let 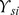 be this button-press count on trial *i* for subject *s*. Because the earning interval was 1 sec, this is simply the work-rate in hertz. Because 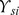 takes non-negative integer values, we assume that it follows a Poisson distribution. We captured within-subject variation in work-rate with the linear model 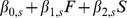. Here 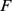 and 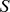 are dummy variables which equal 1 on any female or shape trial, respectively, and equal 0 otherwise. Thus 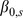 reflects the average work-rate of subject 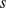 in the presence of male cues. To see this, note that the presence of male cues implies the absence of female/shape cues, i.e. 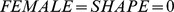, so the equation above yields 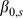. In turn, 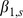 quantifies additive deviations from this (male) baseline due to the presence of female cues. Analogously, 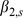 represents deviations from this baseline in the presence of shape cues. To quantify between-subject variation in these effects 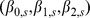 as a function of “reward responsiveness” 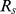, we again used linear regression with the form 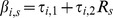. Equation 2 uses standard matrix notation to capture these three, between-subject linear regressions on 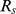


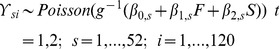
(1)

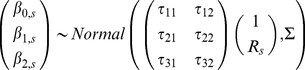
(2)where 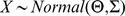 means 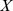 follows a multivariate Gaussian distribution governed by mean 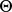 and variance-covariance matrix 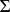, 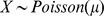 means 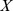 follows a Poisson distribution governed by mean 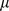 and 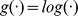 is the ‘canonical’ link function for the Poisson distribution in the context of generalized linear models. The ‘group-level’ parameters 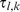 quantify baseline and differential work-rate *on average in the population* and are therefore the object of statistical inference. The estimated 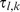 are reported below. This model accommodates subject-wise repeated-measures by affording each subject their own (random) effects [19].

## Results

Subjects button-pressed 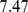 times on average during the earning interval. Statistical inference is based on Equations 1,2. In particular, parameters 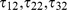 respectively quantify how well reward responsiveness 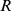 predicts baseline work-rate – in the presence of male cues – and the effect of female and shape cues (relative to male cues). This analysis revealed that self-reported “reward responsiveness” significantly predicted higher baseline work-rate 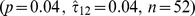, but not their differential work-rate faced with different cues.

Our second question was whether cue-type affected work-rate, independently of subjects' personality. This is quantified by the remaining three parameters, 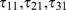. Figure 1 shows how work-rate differed following the presentation of male, female, and shape cues on average over all subjects. By hypothesis (see the introduction), female cues are more invigorating and our subjects should work harder in their presence. In contrast, we observed a statistically significant reduction in work-rate following female cues relative to male cues 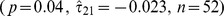. To ask whether work-rate differed between female and shape cues, we simply redefined the baseline condition in Equation 1 to be “shape cues” and re-estimated this model. This revealed no significant difference between work-rate under female-cue versus shape-cue baseline, nor between work-rate under male-cue versus shape-cue baseline.

Because our task and hypotheses directly relate to behavioral reward responsivity, i.e. the tendency for immediate consumable rewards to invigorate behavior, we have focused on BAS II, which operationalizes self-reported reward responsivity. For completeness, we also report post-hoc analysis for the other three sub-scales, BAS I, BIS I, and BIS II. In particular, we re-estimated the model specified in Equations 1,2 three more times, each time replacing the BAS II (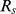) with one of the other sub-scales: BAS I, BIS I, and BIS II. As before, we found a lower work-rate in the presence of female versus male pictures at the 0.05 significance level in every analysis. Also as before, these sub-scales did not predict the effect of erotic reward cues on work-rate at the 0.05 significance level. In contrast to BAS II, BIS I (anxiety) predicted significantly lower baseline work-rate (p = 0.0178). There was a similar trend for BIS II (p = 0.0684), but no observable effect of BAS II (p = 0.691).

## Discussion

We found that subjects with higher self-reported “reward responsiveness” worked harder for money at baseline, but incidental reward cues did not have a greater influence on their work rate. We expected female erotic reward cues to increase work but found that subjects worked about the same under these cues and shape cues: they actually worked less hard under female cues than male cues. This suggests that sexual cues might sometimes have an arresting rather than invigorating action. This is puzzling from the perspective of theories of general motivation or drive [15], [16] or the notion that reward cues might cause a greater urgency to consume anything rewarding [12].

If our effect is indeed attributable to the greater reward value of female cues, it may relate to other literature on reward-dependent performance impairments [20]. This work has proposed various psychological mechanisms to explain the apparent paradox that high reward-motivation can sometimes compromise performance. Most obviously, conscious attention to rewards is thought to disrupt the automatic or overlearned nature of the execution. In classical psychology, “Yerkes-Dodson law” states that either increasing or decreasing motivation beyond an optimal level can compromise learning and performance by affecting arousal [21]. Work from behavioral economics shows that a simple increase in financial incentives can compromise subjects' performance in diverse tasks, including motor learning and cognitive skill [22]. Yet it is important to recall that our task purposefully measured vigor in the absence of such learning or cognitive/executive skill. It remains possible that the lower work-rate on female trials reflected reward-dependent slowing of reaction time [23], meaning that there was less of the one second earning interval left to exploit. Future work should collect specific RTs to explore test this possibility. It is feasible that female images are more salient or distracting, and that this somehow property interferes with subjects' motor response during the effort task, reducing their work-rate. It would be a true testimony to the salience of these images if they could impair performance in a task as cognitively undemanding as ours. Alternatively, the difference that we observed between work-rate under male versus female cues, might reflect a specific motivating feature of *male* cues, either because these muscular images prime exertion or competitiveness. These possibilities should be addressed in future work.

Interestingly, in post-hoc analysis we found that BIS I (anxiety) predicted significantly lower baseline work-rate. We can speculate that anxious subjects were more reluctant to draw attention to themselves by audibly striking the space bar for money and/or were more concerned about damaging the keypad.

**Figure 2:**
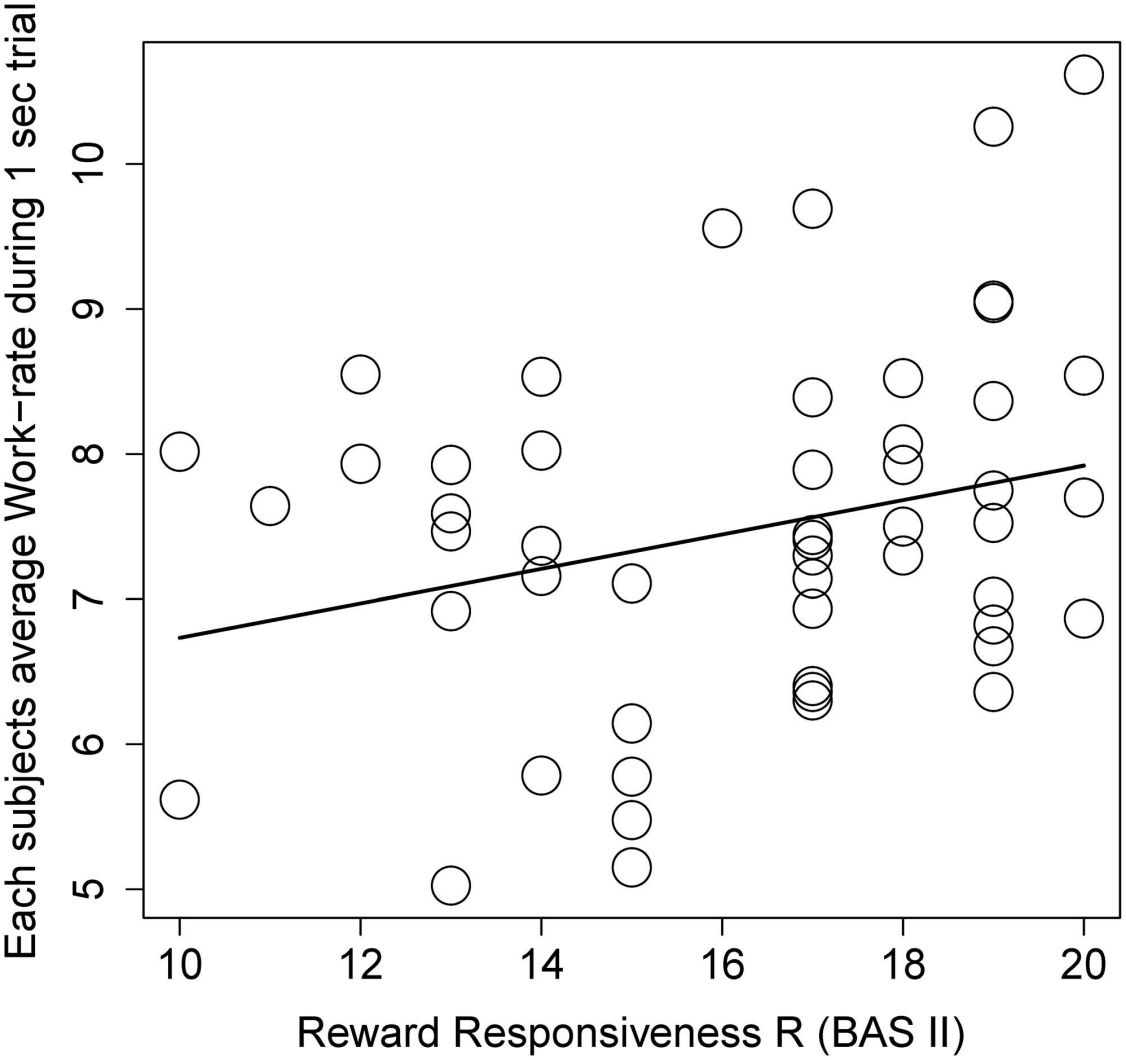
This scatter plot gives the relationship between self-reported reward responsiveness (BAS II) and work-rate, i.e. the number of button presses per one second earning interval.

We now discuss specific features of our task which might limit the generality of our conclusions. First, as Figure 2 illustrates, our task produced relatively low variability in the dependent measure (work-rate), which plausibly reduced statistical power to detect between-trial/subject effects. In retrospect, we believe that a longer “earning interval” might increase this variability, thereby helping us to separate highly motivated conditions/subjects from less motivated conditions/subjects. Second, it is possible that cross-trial generalization effects may obscure trial-specific motivational effects, thereby also reducing our statistical sensitivity. For example, it is possible that images have a temporally sustained impact on behavioral vigor that can obscure trial-by-trial dynamics of vigor [24], [25], [16]. As one reviewer pointed out, this latter possibility might be assessed with an additional between-subject experimental factor, in which subjects perform our effort task in the absence of any cues. This would address two interesting questions: 1) do images affect performance at a contextual level (across trials) and 2) do non-instrumental, contextual motivates differentially based on trait measures like BAS?

Our main result is that self-reported reward-responsiveness predicts the vigor with which subjects pursue instrumental rewards. Paradigms such as ours may have utility in the study of psychiatrically disordered motivation. For example, clinically depressed subjects show substantial impairments in cognitive and motor tasks that require sustained effort [26]. Our task provides one way to assess whether such effects derive from generalized anhedonia or impaired reward responsiveness [27].

## Supporting Information

Materials S1 (PDF)

## References

[1] Gray JA (1990) Brain systems that mediate both emotion and cognition. Cognition & Emotion 4: 269–288.

[2] Smillie LD (2008) What is reinforcement sensitivity? neuroscience paradigms for approach-avoidance process theories of personality. European Journal of Personality 22: 359–384.

[3] Corr P (2004) Reinforcement sensitivity theory and personality. Neuroscience & Biobehavioral Reviews 28: 317–332. 15225974 10.1016/j.neubiorev.2004.01.005

[4] Corr PJ, Pickering AD, Gray JA (1995) Personality and reinforcement in associative and instrumental learning. Personality and Individual Differences 19: 47–71.

[5] McNaughton N, Corr P (2004) A two-dimensional neuropsychology of defense: fear/anxiety and defensive distance. Neuroscience & Biobehavioral Reviews 28: 285–305. 15225972 10.1016/j.neubiorev.2004.03.005

[6] Gray JR, Braver TS, Raichle ME (2002) Integration of emotion and cognition in the lateral prefrontal cortex. Proceedings of the National Academy of Sciences 99: 4115–4120. 10.1073/pnas.062381899 PMC122657 11904454

[7] Scheres A, Sanfey AG (2006) Individual differences in decision making: drive and reward responsiveness affect strategic bargaining in economic games. Behavioral and Brain Functions 2: 35. 17049091 10.1186/1744-9081-2-35 PMC1635418

[8] Franken IH, Muris P (2005) Individual differences in reward sensitivity are related to food craving and relative body weight in healthy women. Appetite 45: 198–201. 15949869 10.1016/j.appet.2005.04.004

[9] Beaver JD, Lawrence AD, van Ditzhuijzen J, Davis MH, Woods A, et al (2006) Individual differences in reward drive predict neural responses to images of food. The Journal of Neuroscience 26: 5160–5166. 16687507 10.1523/JNEUROSCI.0350-06.2006 PMC6674259

[10] Van den Berg I, Franken IH, Muris P (2010) A new scale for measuring reward responsiveness. Frontiers in psychology 1. 10.3389/fpsyg.2010.00239 PMC3153843 21922010

[11] Hartig J, Moosbrugger H (2003) Die “ARES-Skalen” zur Erfassung der individuellen BIS-und BAS-Sensitivität. Zeitschrift für Differentielle und Diagnostische Psychologie 24: 293–310.

[12] Van den Bergh B, Dewitte S, Warlop L (2008) Bikinis instigate generalized impatience in intertemporal choice. Journal of Consumer Research 35: 85–97.

[13] Wilson M, Daly M (2004) Do pretty women inspire men to discount the future? Proceedings of the Royal Society of London Series B: Biological Sciences 271: S177–S179. 15252976 10.1098/rsbl.2003.0134 PMC1810021

[14] Van den Bergh B, Dewitte S (2006) Digit ratio (2d: 4d) moderates the impact of sexual cues on men's decisions in ultimatum games. Proceedings of the Royal Society B: Biological Sciences 273: 2091–2095. 16846918 10.1098/rspb.2006.3550 PMC1635480

[15] Bolles RC, Zeigler HP (1967) Theory of motivation. Harper & Row New York.

[16] Niv Y, Daw N, Dayan P (2006) How fast to work: Response vigor, motivation and tonic dopamine. Advances in neural information processing systems 18: 1019.

[17] Carver C, White T (1994) Behavioral inhibition, behavioral activation, and affective responses to impending reward and punishment: The bis/bas scales. Journal of personality and social psychology 67: 319.

[18] Smillie L, Jackson C, Dalgleish L (2006) Conceptual distinctions among carver and white's (1994) bas scales: A reward-reactivity versus trait impulsivity perspective. Personality and Individual Differences 40: 1039–1050.

[19] Gelman A, Hill J (2007) Data analysis using regression and multilevel/hierarchical models. Cambridge University Press.

[20] Baumeister RF (1984) Choking under pressure: self-consciousness and paradoxical effects of incentives on skillful performance. Journal of personality and social psychology 46: 610. 6707866 10.1037//0022-3514.46.3.610

[21] Sorrentino RM, Short J (1986) Uncertainty orientation, motivation, and cognition. Handbook of motivation and cognition: Foundations of social behavior 1: 379–403.

[22] Ariely D, Gneezy U, Loewenstein G, Mazar N (2009) Large stakes and big mistakes. The Review of Economic Studies 76: 451–469.

[23] Anderson BA, Laurent PA, Yantis S (2011) Value-driven attentional capture. Proceedings of the National Academy of Sciences 108: 10367–10371. 10.1073/pnas.1104047108 PMC3121816 21646524

[24] Chiew KS, Braver TS (2013) Temporal dynamics of motivation-cognitive control interactions revealed by high-resolution pupillometry. Frontiers in psychology 4. 10.3389/fpsyg.2013.00015 PMC3557699 23372557

[25] Kouneiher F, Charron S, Koechlin E (2009) Motivation and cognitive control in the human prefrontal cortex. Nature Neuroscience 12: 939–945. 19503087 10.1038/nn.2321

[26] Cohen RM, Weingartner H, Smallberg SA, Pickar D, Murphy DL (1982) Effort and cognition in depression. Archives of general psychiatry 39: 593–597. 7092490 10.1001/archpsyc.1982.04290050061012

[27] Henriques J, Davidson R (2000) Decreased responsiveness to reward in depression. Cognition & Emotion 14: 711–724.

