# Supplementary material for: Does General Motivation Energize Financial Reward-Seeking Behavior? Evidence from an Effort Task
Source: PLoS One. 2014 Sep 26;9(9):e101936. doi: 10.1371/journal.pone.0101936 (PMC4178010; doi:10.1371/journal.pone.0101936)
Supplement: Materials S1 — (PDF) [file pone.0101936.s001.pdf]

Supplementary Material

Instructions: The following questionnaire consists of a set of statements, which describe oneself. Each statement may apply to you to a different extent ("Disagree", "Rather Disagree", "Rather Agree", and "Agree"). To fill in the questionnaire, please place a cross in the corresponding box. Please answer every statement. Should you feel unsure what to indicate, cross the one answer that suits you the most.

- It gives me a kick when things work out as planned.
- I get frustrated pretty easily if something doesn't work out as I had hoped.
- Even small things make me really happy.
- I become flustered quickly when I realize that I did something wrong.
- I sadden quickly if I don't attain a goal I was aiming for.
- You can barely stop me once I set myself a goal.
- If someone criticizes me, I get insecure and nervous.
- Even little everyday mishaps can really frustrate me.
- The prospect of success energizes me.
- If I feel that what I am doing goes wrong, I get anxious and insecure quickly.
- It stimulates me when I feel that I can attain a personal goal.
- I am easily delighted.
- I can get really sad if something doesn't work out the way I wanted it to.
- Even little mishaps unsettle me considerably.
- It makes me very happy to achieve a goal I strove for.
- I get rather seldom really excited about something.
- If I do something wrong, I immediately fear the consequences.
- Even little mishaps really disappoint me.
- I rarely get excited, even when I get something that I really wanted.
- Even little incentives can strongly motivate me.

0.1. Details about BAS

Regarding internal reliability, Cronbach's alpha for these questionnaire measures are as follows: BIS I (0.84), BIS II (0.79), BAS I (0.74), BAS II (0.75) [9]. Regarding external validity of BAS more generally, previous reports have examined the relation of BAS to affective, para/sympathetic, neurophysiological and behavioral indices. The original publication [6] demonstrated that BAS reward responsiveness predicted positive affect during reward anticipation. More specifically, BAS reward responsiveness has been associated with stronger sexual arousability and excitability [5, 1]. Subsequent imaging literature work has produced evidence that reward responsiveness predicts the strength of blood oxygen level dependent (BOLD) MRI responses to appetitive visual stimuli, including erotic stimuli, within the classical "reward network" [8, 2, 3, 10]. Also relevant to our task is a related paper showing that BAS predicts frontoparietal responses to monetary incentives [7]. Interestingly, in contrast to imaging work, psychophysiological work has shown that BAS reward responsiveness does not correlate strongly with other reliable sympathetic and parasympathetic indices of reward [4]. While BAS reward responsiveness has been related to behavioral performance on some tasks, e.g. the Iowa gambling task, inconsistencies remain in the literature [11]. Furthermore, the relation of these tasks to simple reward responsivity is often unclear. For example, Iowa gambling performance incorporates punishment, reward and learning. Our work was partly motivated by the issue of their external validity: in particular we wanted to ask whether self-reported BAS II can predict behavioral reward responsiveness in a simple task which quantifies energetic responsivity to incidental reward-cues. We quantified this cue-dependent invigoration in terms of its interference on an instrumental task, in which subjects could earn money for each button press.

[1] Anton Aluja and Rafael Torrubia. Hostility-aggressiveness, sensation seeking, and sex hormones in men: re-exploring their relationship. *Neuropsychobiology*, 50(1):102–107, 2004.

[2] César Ávila Rivera, Gabrielle Garbin, Ana Sanjuán Tomás, Cristina Forn Frias, Alfonso Roberto Barrós Loscertales, Juan Carlos Bustamante, Aina Rodríguez Pujadas, Vicente

Belloch, and María Antonia Parcet Ibars. Frontostriatal response to set switching is moderated by reward sensitivity. 2011.

[3] Alfonso Barrós-Loscertales, Noelia Ventura-Campos, Ana Sanjuán-Tomás, Vicente Belloch, Maria-Antònia Parcet, and César Ávila. Behavioral activation system modulation on brain activation during appetitive and aversive stimulus processing. *Social cognitive and affective neuroscience*, 5(1):18–28, 2010.

[4] Sharon L Brenner, Theodore P Beauchaine, and Patrick D Sylvers. A comparison of psychophysiological and self-report measures of bas and bis activation. *Psychophysiology*, 42(1): 108–115, 2005.

[5] Deanna Carpenter, Erick Janssen, Cynthia Graham, Harrie Vorst, and Jelte Wicherts. Women's scores on the sexual inhibition/sexual excitation scales (sis/ses): Gender similarities and differences. *Journal of Sex Research*, 45(1):36–48, 2008.

[6] C.S. Carver and T.L. White. Behavioral inhibition, behavioral activation, and affective responses to impending reward and punishment: The bis/bas scales. *Journal of personality and social psychology*, 67(2):319, 1994.

[7] D. Engelmann and U. Fischbacher. Indirect reciprocity and strategic reputation building in an experimental helping game. *Games and economic behavior*, 67(2):399–407, 2009.

[8] Tim Hahn, Thomas Dresler, Ann-Christine Ehlis, Michael M Plichta, Sebastian Heinzel, Thomas Polak, Klaus-Peter Lesch, Felix Breuer, Peter M Jakob, and Andreas J Fallgatter. Neural response to reward anticipation is modulated by gray's impulsivity. *Neuroimage*, 46(4): 1148–1153, 2009.

[9] Johannes Hartig and Helfried Moosbrugger. Die "ARES-Skalen" zur Erfassung der individuellen BIS-und BAS-Sensitivität. *Zeitschrift für Differentielle und Diagnostische Psychologie*, 24(4): 293–310, 2003.

[10] Hannah S Locke and Todd S Braver. Motivational influences on cognitive control: behavior, brain activation, and individual differences. *Cognitive, Affective, & Behavioral Neuroscience*, 8 (1):99–112, 2008.

[11] Julie A Suhr and John Tsanadis. Affect and personality correlates of the iowa gambling task. *Personality and Individual Differences*, 43(1):27–36, 2007.
